# Digitalization and the Social Lives of Older Adults: Protocol for a Microlongitudinal Study

**DOI:** 10.2196/20306

**Published:** 2020-10-01

**Authors:** Birthe Macdonald, Gizem Hülür

**Affiliations:** 1 URPP Dynamics of Healthy Aging Institute of Psychology University of Zurich Zurich Switzerland; 2 School of Aging Studies University of South Florida Tampa, FL United States

**Keywords:** digitalization, older adults, microlongitudinal, social communication

## Abstract

**Background:**

Digital technologies are increasingly pervading our daily lives. Although older adults started using digital technologies later than other age groups, they are increasingly adopting these technologies, especially with the goal of communicating with others. However, less is known about how online social activities are embedded in older adults’ daily lives, how they complement other (offline) social activities, and how they contribute to social connectedness and well-being.

**Objective:**

Data generated by this project will allow us to understand how older adults use digital communication in their daily lives to communicate with others, how this relates to well-being and social connectedness, and how communication using digital technologies differs from other types of communication depending on situational and individual characteristics.

**Methods:**

Microlongitudinal data were collected from 120 older adults from German-speaking regions of Switzerland to examine these questions. Data collection took place from April 2019 to October 2019. Data collection took place over different time scales, including event-based (reporting all social interactions for 21 days), daily (well-being, loneliness, and technology use every evening for 21 days), hourly (cortisol assessments 6 times per day for 3 days), and baseline (relevant interindividual characteristics, including sociodemographics, health, technology use, personality, and cognitive performance) assessments.

**Results:**

Data collection for this study was completed in November 2019. Participants reported an average of 96.35 interactions across the 21 days. Among the total 11,453 interactions, 5494 (47.97%) were face-to-face, and around 16% each were interactions by phone (1858, 16.16%), email (1858, 16.22%), and text message (1853, 16.18%). Otherwise, 246 (2.15%) of the interactions took place on social media, 96 (0.84)% were letters, and 54 (0.47%) of the interactions took place on videochat.

**Conclusions:**

Participants used a variety of modalities in their daily communication, including digital means such as text messages, email, and video calls. Further analysis will provide more detail as to the role that communication via digital media plays in older adults’ daily lives.

**International Registered Report Identifier (IRRID):**

RR1-10.2196/20306

## Introduction

### Background

Social interactions are a basic human need, serving a multitude of purposes by fulfilling the need for social integration and by providing feelings of closeness, relatedness, social support, and belonging with others [1-7]. The quantity and quality of social interactions have an impact on subjective well-being and quality of life [8,9], and are associated with better health [10-13].

The digital revolution has offered more and more possibilities for individuals to connect with others and share experiences. Younger people use the internet almost universally, while older adults increasingly use digital technology to communicate, albeit at lower proportions than younger adults [14]. Although the use of digital technology comes naturally to those who have been familiar with it their entire lives, or have used the technology during their professional lives, older generations might find it more difficult to become accustomed to using these new technologies. However, these technologies could be more and more useful for older people today. Growing distances between family members as well as declining marriage and birth rates [15,16] may lead to concerns that older adults are at increasing risk of loneliness. This stands in contrast to findings that show lower levels of loneliness in older adults currently compared to previous generations [17,18]. Therefore, it is vital to investigate possible mechanisms by which older adults might compensate for weaker family ties.

One compensatory mechanism is the move from mainly family-oriented to more friend-focused social circles [19,20]. In addition, a greater variety of living situations have become more accepted in recent years; for example, being divorced does not affect social loneliness to the same extent as in previous generations [21]. Finally, the digital revolution offers older adults the opportunity to stay in close contact with family and friends, regardless of geographical distance, as well as the opportunity to connect with new social contacts based on shared interests [22].

Older adults use the internet at increasing rates [14,23]. In Switzerland, 32% of older adults report having a smartphone and 26% own a tablet computer [24]. The internet is used most commonly to communicate with others [25], and the same is true for older adults’ internet use: in Switzerland, 80% of older internet users report using the internet for social interactions [23]. In addition, older internet users agree that the internet has made it easier for them to reach people [26]. Despite the increase in older adults’ internet use, some older people still do not have access to digital technologies. Sociodemographic characteristics play an important role [27], such as education [28] and age [14,23]. Furthermore, cognitive ability is a stronger predictor of older adults’ internet use than age alone [29]. Need for cognition, a personality trait that reflects a preference for cognitively effortful activities, is also positively associated with the frequency of internet use in older adults [30].

Previous research has primarily focused on factors that predict internet use in older adults, as well as the activities older adults perform online. However, relatively little is known about how older adults integrate digital technologies into their day-to-day lives [31]. To begin to understand the role of digital technologies for social interaction and for reducing loneliness in old age, it is vital to investigate the (digital) social lives of older adults.

Information about social interactions is often gathered through retrospective self-reports; however, these responses may be biased by more salient recent events [32]. In contrast, in an event-contingent, microlongitudinal approach, participants answer a set of questions each time a prespecified event, in this case a social interaction, occurs [33]. This enables participants to report their experiences and reactions to this event immediately, or soon after it occurs, and minimizes retrospective bias. Several studies have investigated social interactions using such microlongitudinal designs [34,35]. One study reported an average of 7 interactions that lasted 5 minutes or longer [36,37], highlighting the large amount of data that can be collected using such a design. Thus, detailed information about the social lives of older adults will facilitate the investigation of a variety of research questions related to older adults’ use of digital technology.

### This Study

To study the day-to-day social experiences of older internet users, this study is based on an event-contingent microlongitudinal design. Specifically, participants were asked to complete a brief protocol on every social interaction lasting 5 minutes or longer, and on every text-based interaction (eg, letter, text message, email) over a period of 3 weeks. The brief protocol assessed information about the duration, conversation partner(s), and purpose, as well as the perceived quality of the conversation.

In addition to the interaction reports, participants were asked to fill in questionnaires every evening on their daily mood, health, and feelings of loneliness. At baseline, data on global measures of technology use, cognition, personality, loneliness, and quality of life were collected. Finally, participants were asked to provide 6 saliva samples per day for 3 days, which will be used to examine daily trajectories of diurnal cortisol. Salivary cortisol is a biomarker that is commonly used to assess stress and resilience [38] throughout the lifespan and is also related to loneliness [39]. Salivary cortisol has a detectable daily rhythm, showing high levels in the morning, around 30 minutes after awakening, and decreasing throughout the day [40,41]. This daily rhythm of cortisol secretion can reliably be detected in older adults, despite increased intraindividual variability [42].

### Research Goals

The goal of this study is to understand daily social experiences and their associations with well-being in older internet users. Specifically, the analyses will focus on individual patterns of social communication and their relationship with facets of daily and global well-being, taking into consideration the modalities through which participants communicate.

## Methods

### Participants and Procedure

 A total of 120 community-dwelling participants were recruited for this project. Inclusion criteria were being aged 65 years and above, using digital devices to communicate, sufficient vision and hearing, and being fluent in German. Inclusion criteria were aged 65 years and above, using digital devices to communicate, sufficient vision and hearing, and being fluent in German. 

Participants were recruited via advertisements in local and national newspapers, and through a database of participants hosted at the University of Zurich. Initial contact took place via telephone or email, as preferred by the participant. Eligible participants were invited to a baseline session at the University of Zurich, where they were given detailed information on the study, had the opportunity to ask questions before giving informed consent, received detailed instruction on the study protocol, and received study materials to complete the protocol, including the study smartphones, and Salivettes (Sarstedt AG & Co, Nürnbrecht, Germany) to prepare saliva samples. All information was also provided in written format for participants to take away with them. In addition, they received a phone number to call in case of any questions or problems. The study took place over 21 days starting the day after this initial session. After the first day of the study, participants received a phone call from a research assistant to clarify potential questions. During the study period, a research assistant was on call to answer questions and provide technical support. After 21 days, participants visited the lab to return study materials, give feedback and share their experiences, and to complete a battery of cognitive tests. Participants received CHF 150 (~US $164) to thank them for their participation.

This study protocol was reviewed by the Ethics Committee of the Faculty of Arts and Social Sciences at the University of Zurich (Nr. 19.2.17).

### Design

This study is planned according to a microlongitudinal design with data collection occurring at different time intervals: event-based, time-based, daily, and single assessments at baseline, as summarized in Textbox 1. Participants were required to complete a brief protocol on every spoken social interaction lasting 5 minutes or longer and every text-based social interaction for a period of 21 days. The brief protocol assessed information about the duration, conversation partner(s), purpose, as well as the perceived quality of the conversation. In addition, data on participants’ mood, loneliness, health, and technological issues were collected daily. For the first 3 days of the study period, participants were asked to prepare 6 saliva samples per day. Finally, questionnaires assessing sociodemographic information, health, quality of life, personality, and technology use and attitudes, as well as cognitive ability tests were completed at baseline.

**Data that will be collected throughout the project and timeline of data collection.**

- Event-contingent measures

*What:* Interaction protocol

*When:* To be filled in after each interaction lasting more than 5 min

- Time-based/event-contingent combined (3 days)

*What:* Cortisol

*When:* After waking up, 30 minutes later, 12 pm, 4 pm, 8 pm, Just before going to bed

- Well-being

*What:* Mood, Loneliness, Health, Technical Issues

*When:* Daily

- Individual characteristics

*What:* Personality, Cognitive Performance, Loneliness, Quality of Life, Technology Use

*When:* Baseline

### Apparatus

#### Interaction Data

Participants were given an iPhone 4S at the introductory session at the University of Zurich. The questionnaires were administered with the app “iDialogPad” (G Mutz, Cologne, Germany). This questionnaire was an adaptation of the Rochester Interaction Record [43] and included questions on the time, duration, and communication medium of the interaction; the purpose of the interaction and the interaction partner; as well as affective states and interpersonal behavior during and after the interaction (the full questionnaire can be accessed on the Open Science Framework platform [44]). Participants were asked to record interactions based on spoken conversations lasting longer than 5 minutes and any text-based conversation (eg, letters, emails, text messages). For conversations by text message, we asked participants to immediately record any text message they sent or received, unless they were aware from the beginning that there would be a longer exchange, in which case they could record this as one conversation, and indicate how many text messages each person sent or received.

#### Daily Well-Being

Participants filled in the Positive and Negative Affect Scale [45] each evening, with the addition of 4 items (2 positive and 2 negative) assessing loneliness. They also indicated the state of their health and physical well-being, as well as whether they had experienced any problems with technology (not necessarily related to social communication) that day. If they encountered such a problem, they were asked to describe it briefly using a few words. A daily reminder on the study smartphone reminded them to complete this questionnaire.

#### Cortisol

Participants were asked to provide a total of 18 saliva samples for 3 days (6 samples per day). The samples were prepared using Salivettes, which were labeled with the day and time they were intended to be used and the participant number. Participants were instructed to provide the first sample of each day before getting out of bed in the morning; the second sample 30 minutes later; the third, fourth, and fifth samples at 12 pm, 4 pm, and 8 pm, respectively; and the sixth sample just before going to bed. They were reminded by notifications on the study smartphone to collect each sample. Participants were asked not to eat, drink, smoke, or do any exercise for 30 minutes before providing each sample. Along with each sample, they were asked about their activities during the 30 minutes prior to providing the sample. During the initial session at the University of Zurich, research assistants explained exactly how to prepare the saliva samples using the Salivettes, and gave the participants the opportunity to try this out.

Participants were instructed to keep the samples in the freezer before bringing them to a final session at the University of Zurich. They were supplied with freezer elements to keep the samples cool during transport back to the university after the data collection period. Saliva samples were stored in a freezer until they were analyzed at the laboratory of the Clinical Psychology and Psychotherapy unit at the University of Zurich.

#### Interaction Partners

In the interaction questionnaire, participants were asked to report who they were interacting with by assigning a set of initials or another identifier to each interaction partner. To be able to remember the identifiers, participants were provided a form to note down the information. This form was not collected from participants to protect the identity of the interaction partners.

At the final session at the University of Zurich, participants were asked to fill in a short questionnaire asking about their relationship with each interaction partner. This also included questions about the age of the interaction partner as well as the participant’s relationship to them, how long they had known each interaction partner, and their spatial distance. In addition, participants provided information about their social network using the hierarchical mapping technique [46]. Participants indicated how close they felt to each interaction partner and also included any other individuals whom they consider to be part of their social circle but who they were not in touch with during the study period. The full codebook can be accessed on Open Science Framework [44].

#### Baseline Measures

Participants filled in a set of questionnaires in their own time during the study period. They were given a choice between filling in an online version of the questionnaire that they could access through an emailed link or a pen-and-paper version that they could take home with them at the initial study session. The questionnaire included sociodemographic information, technology use [47-51], communication preferences, concerns related to internet use, social network [52,53], loneliness [54], well-being [45,55], health [56,57], and personality [1,58-64]. The full codebook can be accessed on Open Science Framework [44].

#### Cognitive Measures

Participants completed a battery of cognitive tests at the final session, including measures of reasoning (subtests of the Berlin Intelligence Structure test: numerical reasoning, delayed recall, numerical/logical reasoning, verbal reasoning/general knowledge, spatial reasoning, verbal reasoning, numerical reasoning/memory [65]) and the Leistungsprüfsystem für 50-90-jährige (LPS 50+; subtest 3, spatial reasoning [66], perceptual speed [67,68], vocabulary [69]).

## Results

### Sample Demographics and Descriptive Characteristics

Data collection started in April 2019 and was completed in November 2019. Participants were on average 73 years old (SD 5.08, range 66-95 years). Most of the participants were highly educated: 26.7% (32/120) completed their school education with the Swiss *Matura*, a secondary school degree qualifying for university education. The vast majority (98.3%, 118/120) completed further training after school with 22.5% (27/120) completing university, including 4.2% (5/120) participants with a PhD.

In general, participants were very technologically versed. On the computer proficiency questionnaire [47], they scored an average of 132 out of 160 possible points; 96.7% (116/120) of participants owned a computer, 86.7% (104/120) owned a smartphone, and 65.8% (79/120) owned a tablet. Generally, 40.0% (48/120) of participants reported that using the internet to communicate had made them feel closer to other people, 50.0% (60/120) reported that nothing had changed, and 10.0% (12/120) reported that they felt less close to other people.

Participants reported an average of 96.35 interactions across the 21 days (SD 67.57, range 9-517). These data are summarized Table 1. Women reported a higher average of interactions than men. The majority of interactions were face-to-face, followed by interactions by phone, email, and text message with relatively equal frequencies. Social media, letters, and videochat were the least frequent types of interactions reported.

Most conversations took place with people the participants knew, with less than 10% of the conversations taking place with people they did not know and service providers, respectively.

**Table 1:** Descriptive characteristics of the reported conversations (N=11,453).

Characteristic		Value
**Number of conversations reported (mean)**		
	Total	96.35
	Women	98
	Men	71
**Conversation modality, n (%)**		
	Face to Face	5494 (47.97)
	Phone	1851 (16.16)
	Email	1858 (16.22)
	Text	1853 (16.18)
	Social media	246 (2.15)
	Letters	96 (0.84)
	Videochat	54 (0.47)
**Conversation partner, n (%)**		
	Known to the participant	9700 (84.69)
	Unknown to the participant	936 (8.17)
	Service providers	817 (7.13)

### Examples of Planned Analyses

These data will enable more detailed investigation of how daily social interactions can shape older adults’ daily lives, as well as how intraindividual interaction patterns are related to individual differences such as personality or cognition. Specifically, we will also be able to further examine the role of different patterns of communication through digital modalities in older adults’ daily lives and well-being. To analyze the interplay between (digital) social communication, well-being, and individual differences, we will conduct multilevel analyses to capture the individual variety in communication patterns. This includes investigating the relationship between daily communication patterns and mood in relation to personality and closeness with interaction partners, the relationship between personality (especially openness) and use of digital technologies, and the relationships among daily communication patterns, loneliness, and diurnal cortisol.

## Discussion

With the increasing digitalization of our society, communication using digital technologies is becoming more prevalent. Older adults have also adopted these technologies and are starting to use them widely. The data generated by this project will allow us to understand how older adults are using digital communication technologies in their day-to-day lives, and how the use of these technologies is related to social connectedness and well-being. Specifically, one of the major aims of our study is to understand the role of the quantity and quality of social interaction for social connectedness and well-being, and differences between communication media in this association. Furthermore, we will examine the role of situational and individual characteristics. Overall, the available data will allow us to examine these questions in rich detail.
